# Associations between *Cryptococcus* Genotypes, Phenotypes, and Clinical Parameters of Human Disease: A Review

**DOI:** 10.3390/jof7040260

**Published:** 2021-03-30

**Authors:** Marhiah C. Montoya, Paul M. Magwene, John R. Perfect

**Affiliations:** 1 Division of Infectious Diseases, Department of Medicine, Duke University, Durham, NC 27710, USA; Marhiah.Montoya@Duke.edu; 2 Division of Infectious Diseases, Department of Molecular Genetics and Microbiology, Duke University, Durham, NC 27710, USA; 3 Department of Biology, Duke University, Durham, NC 27710, USA; Paul.Magwene@Duke.edu

**Keywords:** *Cryptococcus*, genotype, phenotype, virulence, cryptococcal meningitis, pulmonary cryptococcosis, clinical presentation, clinical outcomes

## Abstract

The genus *Cryptococcus* contains two primary species complexes that are significant opportunistic human fungal pathogens: *C. neoformans* and C*. gattii*. In humans, cryptococcosis can manifest in many ways, but most often results in either pulmonary or central nervous system disease. Patients with cryptococcosis can display a variety of symptoms on a spectrum of severity because of the interaction between yeast and host. The bulk of our knowledge regarding *Cryptococcus* and the mechanisms of disease stem from in vitro experiments and in vivo animal models that make a fair attempt, but do not recapitulate the conditions inside the human host. To better understand the dynamics of initiation and progression in cryptococcal disease, it is important to study the genetic and phenotypic differences in the context of human infection to identify the human and fungal risk factors that contribute to pathogenesis and poor clinical outcomes. In this review, we summarize the current understanding of the different clinical presentations and health outcomes that are associated with pathogenicity and virulence of cryptococcal strains with respect to specific genotypes and phenotypes.

## 1. Introduction

The globally ubiquitous basidiomycete, *Cryptococcus* spp., is an environmental yeast capable of causing disease in humans [1]. In nature, the ecological niche of this fungus is primarily within the bark or hollows of trees, decaying wood, bird guano, soil and other organic matter [2,3,4]. Many of the evolutionary adaptations and stress-induced compensatory mechanisms that have equipped Cryptococcus neoformans and Cryptococcus gattii to be environmentally resilient likely contribute to their success as human pathogens in immunocompromised populations and, less commonly, in immunocompetent populations [2]. Cryptococcal disease represents a dynamic “two-way street” interaction between host and yeast. Immunocompromising conditions such as HIV/AIDS, solid organ transplant, liver disease, lupus, certain cancers and cancer therapies, and corticosteroid use are major risk factors for cryptococcosis. Furthermore, immunocompetent hosts may also have unknown immunological perturbations such as idiopathic CD4+ T cell lymphocytopenia, anti-GM-CSF antibodies, or other genetic attributes that predispose them to cryptococcosis [5,6]. Without accounting for pulmonary cryptococcal infections or including other patient populations, it is estimated that cryptococcal central nervous system (CNS) infections cause >180,000 deaths per year globally in the HIV-positive population alone [7].

**Figure 1:**
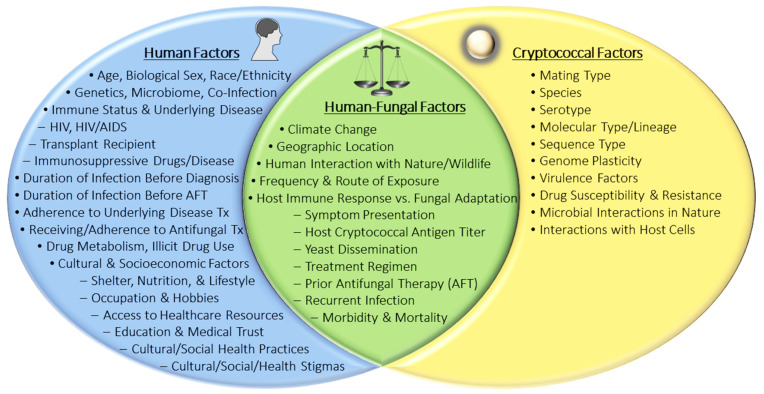
Factors that Contribute to Cryptococcosis Infection and Outcome. Abbreviations: Antifungal Therapy (AFT), Treatment (Tx).

Most exposures begin with inhalation of infectious cryptococcal propagules (e.g., spores and/or yeasts) from the environment into the lungs where the yeast can be cleared by the immune system or reside dormant, establishing pulmonary colonization or lymph node complexes [2,4,8,9,10]. The timing of exposure may vary by geographic region and may depend on other socio-cultural factors, but by adulthood, approximately 70% of people have developed antibodies to Cryptococcus [11,12,13]. Once inside the human host, the traits that contribute to the success of *Cryptococcus* in the natural environment may act as virulence factors that contribute to fungal survival, disease initiation, and progression of infection. Extensively characterized in vitro, the classic cryptococcal virulence factors include the polysaccharide capsule, melanin formation, growth at host body temperature, and secretion of enzymes such as phospholipase, laccase, and urease [1,2]. Successful disease initiation and progression likely rely on numerous genotypic and phenotypic factors of both the host and the fungus (Figure 1). More simply, a host must be susceptible and exposed to a cryptococcal strain that is sufficiently pathogenic before disease can occur. Susceptible colonized hosts may experience an asymptomatic latent pulmonary infection that can become active pulmonary cryptococcosis (PC) or disseminate throughout the body to the CNS causing cryptococcal meningitis (CM) during an immunosuppressive event [8,11,13,14]. In hosts that are susceptible upon exposure to the yeast, acute infection may manifest and disseminate without a dormant stage. In general, *Cryptococcus* preferentially localizes to the lungs and brain during infection; however, most organs have been reported as either primary sites of infection (e.g., skin) or secondary sites as a result of dissemination [15,16,17,18].

To develop better cryptococcosis prevention and treatment methods, we must first identify and understand the human-yeast phenotypic and genotypic factors that contribute to disease and outcome (Figure 1). Historically, cryptococcal genetics and genomics have been studied to understand how *Cryptococcus* species and strains transitioned from an environmental yeast to human pathogen [19]. From polymerase chain reactions (PCR) and Sanger sequencing to multi-locus sequence typing (MLST), whole genome sequencing (WGS), and quantitative trait loci (QTL) mapping, these molecular methods have been instrumental in studying the genetic differences between cryptococcal species. Moreover, these methods have identified distinct genetic factors that contribute to their pathogenicity and varying virulence phenotypes. In vitro experiments and in vivo cryptococcosis animal models have provided a wealth of information regarding the disease capabilities of both environmental and clinical isolates. Experimental phenotyping has also shown that environmental and clinical isolates are both generally equipped with the same classic virulence attributes; however, not all environmental isolates can establish infection in mammals or can disseminate from the lungs to the CNS [20,21]. Furthermore, among pathogenic cryptococcal strains, virulence severity can vary, as can disease presentations [22]. These observations suggest: (1) the classical virulence factors discovered to date contribute to, but may not be solely sufficient for, full pathogenicity in mammals; (2) there may be undiscovered phenotypic and/or genotypic attributes that contribute to pathogenicity and/or virulence; and (3) the cryptococcal genotypic/phenotypic attributes required for pathogenicity and virulence may vary depending on the host genotype/phenotype. Technological advances and growing interest in genome-wide association studies (GWAS) have opened the door to discover novel associations between genomic variations, virulence phenotypes, and clinical outcomes. Using these traits, comparative studies can identify species and strain-specific differences associated with clinical parameters of human disease.

To further understand the dynamics of cryptococcal colonization, disease initiation, and progression that impact clinical manifestations and health outcomes, it is important to study the host-pathogen relationship in the context of human infection. Thus, we will first review the science regarding important host-yeast factors that contribute to cryptococcosis and then summarize the known associations between the clinical parameters of human disease and cryptococcal genotypes and observed fungal phenotypes (Figure 1).

## 2. *Cryptococcus* Genotypes and Virulence Phenotypes

The heterogeneous genus, *Cryptococcus*, includes over 70 different species; though few are human fungal pathogens [23]. Widespread sequencing and recent molecular phylogenetic analyses have led to the designation of new species and lineages within the genus, and the taxonomy of the group as a whole is still in flux. Members of the *C. neoformans* and *C. gattii* species complex are the primary etiologic agents of human cryptococcosis. In addition to utilizing molecular methods for strain differentiation, the characterization of cryptococcal virulence phenotypes often involves in vitro experiments and the use of different induction media to elicit expression of a specific virulence attribute. In this section we will describe the distinct cryptococcal genotypic designations and will summarize the known virulence mechanisms of cryptococcal phenotypes that have been associated with human disease.

### 2.1. Cryptococcus Genetics and Genotypic Descriptors

**Figure 2:**
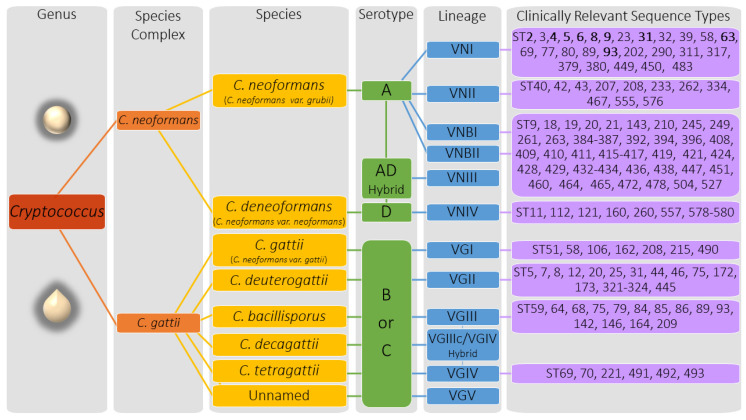
The *Cryptococcus* species complex. Cryptococcal strains are distinguished by species complex, species, serotype, molecular type or lineage, and sequence type. **Bold** text indicates the Sequence Types that are observed more frequently among clinical isolates (i.e., ST2, ST4-ST9, ST31, ST63, ST93) in certain geographical regions. Genus panel shows the difference in cryptococcal cell shapes that occur in each species complex; *C. neoformans* strains are often round in shape and *C. gattii* strains can have an oval to teardrop shaped morphology [2].

The molecular age of taxonomy for the pathogenic cryptococcal species has dramatically divided the groups into sibling species or clades through genetic relationships. Within the C. neoformans and C. gattii species complex, twelve molecular types or lineages have been recognized in the literature (Figure 2). Strains within these species complexes are mostly haploid with genomes that range in size of ~16 to 19 Mb, contain ~14 chromosomes, have a bipolar mating system encoded by the MAT locus that can be distinguished as either MATa or MATα using molecular methods (e.g., PCR, sequence analysis, etc.) or by using a mating assay (Table 1). A major structural feature of Cryptococcus strains, the polysaccharide capsule, exhibits strain-specific antigens resulting in distinct “serotypes” designated A, B, C, or D; though AD, AB, and BD hybrids have also been found. Serotypes can be distinguished using molecular methods (e.g., PCR, MLST, WGS, etc.) or by phenotypic characteristics such as cell morphology (sexual forms), growth differences on media containing different components (carbon/nitrogen sources, drug/chemicals, etc.), or agglutination reactions against capsular antigens.

The C. neoformans species complex includes the species C. neoformans (formerly C. neoformans var. grubii) and C. deneoformans (formerly C. neoformans var. neoformans) (Figure 2). The C. neoformans species branch includes serotype A strains and the C. deneoformans lineage includes serotype D strains. C. neoformans is further divided into genetically distinct sub-lineages with the designations VNI, VNII, VNBI, and VNBII. The VNIII, AD serotype is a hybrid of C. neoformans VNI (serotype A) and C. deneoformans VNIV (serotype D) [3,23,24]. The C. gattii species complex has been divided into several sibling species that exhibit serotypes B or C including C. gattii (VGI), C. deuterogattii (VGII with subtypes VGIIa, VGIIb, VGIIc), C. bacillisporus (VGIII), C. tetragattii (VGIV), and the hybrid species C. decagattii (VGIIc/VGIV). The C. gattii species complex continues to expand with the recently discovered unnamed cryptococcal species that has added the new VGV lineage to the complex [25].

Before whole genome sequencing was common, early genetic studies utilized MLST to differentiate *C. neoformans* and *C. gattii* isolates into sequence types (ST) based on the genetic sequences of seven genes (CAP59, GPD1, IGS1, LAC1, PLB1, SOD1, and URA5) [26]. These MLST studies found that sequence types tend to cluster by geographic location and, like the other genotypic descriptors, have been associated with cryptococcal virulence capabilities and health outcomes. Sequence types may be imperfect reflections of the genetic variation and relationship between strains in comparison to WGS, however, these designations have identified important associations between yeast and host.

### 2.2. Cryptococcus Virulence Phenotypes

In nature, *Cryptococcus* utilizes its virulence factors which include the polysaccharide capsule, melanin formation, and secretion of enzymes such as phospholipase, laccase, and urease to survive the ever changing environmental conditions [1,2]. These virulence factors are regulated by stress-signaling pathways such as the high osmolarity glycerol (HOG) pathway, Ras/cyclic AMP/protein kinase A (Ras/cAMP/PKA) pathway, calcineurin-calmodulin pathway, Mpk1 pathway, target of rapamycin (TOR pathway), unfolded protein response (UPR) pathway, and Rim101 pathway. In addition to activation of virulence factors, these stress-signaling pathways induce a variety of reproductive and metabolic changes. In the context of disease, all of these changes contribute to the ability of the yeast to survive at host temperatures, which is essential for establishing mammalian infections. Moreover, these stress-signaling pathways can either contribute to the survival of an individual cryptococcal clonal population or induce genetic alterations that may allow for the survival of future fungal progeny. With much of our knowledge having been derived from in vitro experiments or nonhuman disease models, the classic cryptococcal virulence phenotypes continue to be investigated and have been reviewed in detail [2,20,38,39]. Before discussing the role of virulence factors/phenotypes in affecting various clinical parameters of human cryptococcosis, it is important to define the distinct roles that known virulence factors play in the context of human colonization and infection.

#### 2.2.1. Capsule

The polysaccharide capsule is a complex and dynamic structure that is important for survival within the host and for pathogenicity [38,39,40,41]. *Cryptococcus* can adapt to external stresses and environmental signals such as nutrient availability, pH, osmotic stress, temperature, atmospheric oxygen or carbon dioxide (CO_2_) concentrations, antifungal drugs, and signals related to host biology (e.g., serum, immune cells, infection site, etc.). In response to environmental conditions, *Cryptococcus* can initiate the expansion of its polysaccharide capsule and alter the capsule composition. The capsule dynamically changes in structure, density, and size to optimize chances of survival [2,40]. Structurally, the capsule is primarily comprised of glucuronoxylomannan (GXM), glucuronoxylomannogalactan (GXMGal), and mannoproteins [38,39]. Variation in the structure and proportion of each component within the capsule will change its antigenic properties which are used to distinguish between the different serotypes [39]. Both GXM and GXMGal are important for survival at host temperatures (thermotolerance) and can contribute to adhesion to host endothelial cells [20].

Though there have been conflicting results in the attempt to precisely correlate capsule size with virulence in mammals, it is clear that the capsule contributes to both protection against and evasion of the mammalian immune system [42,43,44,45,46]. In mammalian hosts, capsule formation begins in the early stages of the infection process and is fundamental to establishing disease to the point that acapsular cryptococcal strains have been shown to be avirulent in cryptococcosis mouse models [47,48,49,50]. Changing the size and density of the capsule affects the ability of host macrophages to fully ingest the fungal cell and conceals the cell wall epitopes needed for binding [39]. If *Cryptococcus* is ingested by immune cells, the capsule protects against reactive oxygen species and degradation by enlarging to prevent host enzymes from reaching the cell wall or by shedding capsule to the point that the immune cell ruptures [51,52]. In addition, *Cryptococcus* can shed parts of the capsule at the site of macrophage interaction to prevent whole cell phagocytosis. Successful survival and replication within macrophages aids in pathogenesis and dissemination throughout the body [1]. Furthermore, the capsule plays a central role in disease diagnostics through the detection of polysaccharide capsular antigens in patient samples and clear visual distinction by microscopy from other pathogenic yeast.

#### 2.2.2. Cell Wall and Melanin

In addition to employing capsule formation as a defense against immune cells and the changing environments within compartments of the human host, the cell wall can also adapt by altering the proportions and arrangements of α-glucans, β-glucans, chitin, chitosan, glycosylphosphatidylinositol (GPI)-anchored proteins and other components such as sialic acids [53]. Changing the structure and density of the cell wall in conjunction with inducing melanin and capsule formation is thought to inhibit ease of penetration into the cryptococcal cell by host immune cells and antifungals. Like the capsule, melanin formation is an inducible virulence phenotype that has been well characterized both in vitro and in animal models [54]. Though melanin formation is not essential for establishing human infection, it does contribute to thermotolerance and may contribute to counteracting the effects of free radicals produced by immune response while also reducing sensitivity to antifungals during therapy [54]. Outside of melanin formation, the enzyme laccase has been implicated to have a novel virulence activity related to its utilization for nonlytic exocytosis from macrophages [55].

#### 2.2.3. Secretion of Degradative Enzymes

Secretion of active extracellular enzymes such as proteases, phospholipases, and urease contribute to virulence by damaging and degrading host molecules as a means to acquire nutrients, combat immune response, and disseminate throughout the body [56,57,58]. Lack of these enzymes in mutants has been associated with reduced virulence, but not complete loss of pathogenicity in mammalian hosts. The association between the secretion of these enzymes and virulence in humans still requires further investigation, but animal models have provided in vivo insight regarding their role in disease. Phospholipases enhance adhesion to lung epithelial cells and hydrolyze phospholipid ester linkages to penetrate host tissues [59,60]. A murine model using endotracheal inoculation or intravenous tail vein injection of *Cryptococcus* found that phospholipase B was required for interstitial tissue growth and efficient dissemination from the lung to the brain but not required for crossing the blood-brain barrier [57,61,62]. Urease hydrolyzes urea to produce ammonia and carbamate, a tool that is helpful in regulating environmental pH and can damage host cell membranes. Intravenous tail-vein injection and inhalational nares inoculation in murine models showed that urease-positive strains resulted in reduced survival of mice as well as increased dissemination to the brain in comparison to urease-negative strains [58,63]. In the rabbit cryptococcal meningitis model, intracisternal injection (direct brain injection into cisterna magna) found that both urease-negative and urease-positive cryptococcal strains were equally virulent [58,63].

#### 2.2.4. Novel Virulence Phenotype: Yeast Cell Size

Recent studies have identified associations between yeast cell size and virulence. Currently, there are three suggested cryptococcal yeast cell sizes (categorized by diameter length) that have been associated with virulence: (1) Average cell ~4–7 µm; (2) Micro cell ≤ 1 µm; (3) Giant or Titan cell ≥11 µm with some as large as 100 µm. Each of these cell sizes have been observed in human isolates and recapitulated in murine cryptococcosis models [22,64,65]. In addition to their cell size, both Titan and Micro cells have different attributes that contribute to virulence and disease manifestation. Titan cells are polyploid which has been suggested to be useful to overcome environmental or antifungal stress by producing better suited progeny that are haploid, diploid, or aneuploid [66]. In murine models, this large cell phenotype is accompanied by changes in the capsule structure and cell wall component proportions that increase its thickness which contribute to cryptococcal virulence by affecting recognition and phagocytosis by the host immune system [67,68,69]. In murine pulmonary models, Titan cells primarily localize to the lung where its attributes are thought to aid in survival and pathogenesis while Micro cells are proposed to be better equipped for dissemination and can localize efficiently in the brain [70,71]. The phenotypic attributes that contribute to virulence of Micro cells include increased capsule shedding which effects phagocytosis of immune cells and hampers immune response by preventing T-cell proliferation and proinflammatory cytokine production [44]. Much is unknown about how cell size contributes to pathogenicity and virulence in humans, but these initial findings provide insight into the important phenotypic characteristics for yeast survival at different infection sites.

## 3. Cryptococcosis Clinical Diagnostics and Measures of Disease Severity in Humans

### 3.1. Clinical Diagnostics to Detect Cryptococcus in Humans

Timely and accurate diagnosis of cryptococcal disease can impact patient morbidity and mortality. The two primary cryptococcal diagnostic methods are visual identification of *Cryptococcus* by microscopy and detection of capsular antigen. The three most common types of clinical specimens used to detect presence of *Cryptococcus* are neurological, hematological, or respiratory. Neurological specimens are generally either cerebrospinal fluid (CSF) or brain tissue. Hematological specimens can include whole blood, serum, or plasma. Respiratory specimens commonly are from lung biopsy, bronchoalveolar lavage (BAL) fluids, and sputum. The history and evolution of cryptococcal clinical diagnostics has previously been reviewed so, in this section, we will summarize the main diagnostic techniques currently used in both resource-rich and resource-limited regions [72,73,74,75].

#### 3.1.1. Cell Culture, India Ink Staining, and Quantitative Yeast Counts

Visual identification of cryptococcal yeast cells has historically been the standard in clinical testing and diagnosis of cryptococcosis. The current visual methods that are routinely used in clinical laboratories involve culturing yeast cells from patient specimen and/or staining with India Ink to visualize the distinct “halo-like” polysaccharide capsule around the yeast cell by microscopy. Each of these methods have their own advantages and disadvantages, however culturing methods are less sensitive than capsule antigen detection because they heavily rely on cryptococcal fungal burden in the patient specimen.

India Ink staining is a rapid diagnostic test that requires minimal training and can be done in both resource-rich and resource-limited settings. This test is most often done using patient CSF which limits its usage to patients with CM [74]. The test consists of mixing equal parts of CSF and India Ink on a slide then visualizing the yeast by microscopy. Unfortunately, the sensitivity of India Ink staining ranges from 30–86% and can vary depending on the patient’s immune status and the fungal burden due to its lower limit of detection (10^3^–10^4^ yeast cells/mL of CSF) [74,76,77,78,79]. The lower limit of detection is a significant drawback because it can provide false negative results for patients tested during the early stages of disease. False negative results combined with nonspecific cryptococcosis symptoms can lead to misdiagnosis, progression of cryptococcosis, and death. Regardless, India Ink staining remains an integral diagnostic method for CM, especially in resource-limited regions.

Culturing *Cryptococcus* requires greater resources, laboratory infrastructure, and training. CSF and blood are the common specimens used for cultures. This diagnostic method consists of plating patient specimen onto solid media and waiting for yeast colonies to form during incubation. The solid media used in this diagnostic test generally contains chemicals that produce fungal-specific color change or are selective in such a way that promotes fungal growth while inhibiting bacterial growth (e.g., Sabouraud’s dextrose agar (SDA), potato dextrose agar (PDA), bird seed agar, or Mycosel agar). Obtaining conclusive results from culturing can take anywhere from two days to two weeks depending on a variety of factors such as local resources, patient fungal burden, and presence of co-infecting or contaminating organisms [79]. Like India Ink staining, the culturing method also has reduced sensitivity which can vary by the type of solid media that is used [80].

The last yeast visualization method, quantitative yeast counts, is used to determine fungal burden in patients by plating serial dilutions of CSF on solid media such as SDA, incubating to allow for fungal growth, and counting the resulting colonies to calculate colony forming units per milliliter (CFU/mL) of CSF. Quantitative yeast counts are an uncommon practice in the clinical setting and are generally only done as part of research efforts. There are conflicting reports regarding the association between fungal burden and mortality or persistent infection [81,82,83]. However, as we will discuss in the following sections, these conflicts may be due to patient characteristics such as prior drug exposures such as antiretroviral therapy (ART) medications (e.g., ART-naïve versus ART-exposed) or due to cryptococcal strain-specific immunological responses. In addition, quantitative yeast counts can be done from initial diagnosis and throughout antifungal therapy (AFT) to determine the linear rate of fungal clearance (log_10_CFU/mL CSF), a measurement called early fungicidal activity (EFA). Like fungal burden, EFA, has also been inconclusively associated with therapeutic response and all-cause mortality but some evidence suggests EFA may correlate with time-specific mortality [84].

#### 3.1.2. Cryptococcus Antigen

Detecting cryptococcal antigen (CrAg) in patient CSF, serum, or plasma is done using sensitive assays including the enzyme immunoassay (EIA) (>90% sensitive), latex agglutination (LA) assay (>90% sensitive), and lateral flow assay (LFA) (>95% sensitive), [75,77,85]. CrAg tests are affordable (especially LFA), allow for early diagnosis in asymptomatic patients, and are considerably more rapid [86]. The LA and EIA CrAg diagnostic assays require an extensive laboratory infrastructure with reliable utilities (electricity, refrigeration, etc.), more training to perform the assays, and an established economic supply chain.

EIA CrAg tests begin with the addition of a positive control solution, negative control solution, and patient samples into individual microwells that are coated with anticryptococcal capsule polyclonal antibodies, an incubation step, and wash step to remove anything that did not bind to the antibody coated wells. Then, an enzyme conjugate solution is added and, if cryptococcal antigens are present, the antigens bind the antibody-enzyme conjugate during an incubation step. After incubation, the microwells are washed to remove unbound conjugate and a substrate solution is added to the wells, incubated, and finally a stop solution is added to stop the enzymatic reaction. Th EIA CrAg test results in a colorimetric change that varies in color intensity depending on the amount of bound enzyme. The results can be read visually or by spectroscopy.

In LA CrAg tests, patient samples are first processed according to the assay manufacturer instructions, spotted onto the antibody-coated latex beads, arranged on a slide, and incubated until the reaction is terminated. The results are read by visual inspection. If the sample is positive for CrAg, white clumps will aggregate in the test area which can be interpreted on a scale from 1+ to 4+. LA assays are currently available but require skilled laboratory workers with more extensive training and are more expensive compared to the LFA which is the assay that is currently most widely used.

Instead of incubating the patient’s processed CSF or serum/plasma on a slide, the LFA is a dipstick sandwich immune-chromatographic assay that utilizes capillary flow by moving the patient sample from a reservoir (e.g., microtube) through the conjugate pad (binds patient CrAg to a detection antibody), the test line (nitrocellulose membrane with immobilized anti-cryptococcal antibodies binds patient CrAg), and the control line (binds detection tag). Much like reading a common pregnancy test, bands will appear on both the test line and control line for a positive test while only a control line will appear for a negative test. LFA strips cost around $2–$4 per test and do not require copious amounts of patient specimen for a reliable test. In addition, LFA is the most rapid CrAg test.

Aside from providing qualitative test results, CrAg assays can also provide quantitative results in the form of a CrAg titer. To determine the CrAg titer, both LA and LFA can be conducted using multiple 1:2 serial dilutions of the patient specimen. The CrAg titer is defined as the dilution with the last positive test. Like quantitative yeast counts and EFA, CrAg titers (>1:160) have been associated with mortality [87]. In addition, serum CrAg titer in HIV-infected patients is predictive of cryptococcal disease progression. Specifically, HIV-infected patients with serum/plasma CrAg titers ≤ 1:80 have reduced probability of CNS involvement while those with CrAg titers ranging from 1:160–1:640 have greater probability of CNS involvement [75,88,89]. Additionally, CrAg titers ≥ 1:1280 are indicative of active CM. As a further benefit, CrAg tests have a demonstrated potential to be a useful tool in broad scale public health efforts to aid in early diagnosis and treatment of cryptococcal infections by screening at-risk persons in areas where there is high incidence of HIV and cryptococcal disease; thereby reducing mortality [90,91,92].

### 3.2. Human Clinical Phenotypes: Clinical Measures of Disease Severity

Epidemiological trends of cryptococcal infections have identified differences in patient susceptibility, disease presentation, and outcome depending on human or cryptococcal genotypes and phenotypes. For this review, human clinical phenotypes (human phenotypes) will include drug exposure, underlying conditions (though we will indicate underlying conditions with a genetic component), outcome (morbidity, mortality, disability), and clinical measures of disease severity to include symptoms, radiology, and the values of clinical diagnostic tests (e.g., opening pressure, HIV-viral load, hematological cell counts, antigen presentation, etc.). Human genotypes will include biological sex, race/ethnicity, and human genetic attributes.

#### Symptoms, Radiology, and Clinical Diagnostics

Signs and symptoms of cryptococcosis can be categorized as neurological, pulmonary, or immunological. Common symptoms include cough, dyspnea, headache, neck stiffness or pain, visual acuity changes, light sensitivity, auditory changes, altered mental status (e.g., dizziness, confusion, behavioral changes, dementia-like symptoms, lethargy, coma), seizures, vomiting, and fever. Interestingly, some patients that have pulmonary cryptococcosis infections may not display symptoms despite having an active infection that can potentially disseminate to the brain. In such cases, patients may not be tested for cryptococcosis until dissemination occurs and CM symptoms appear. In resource-rich areas with established healthcare infrastructures, radiology can be used for cryptococcosis diagnosis and to assess disease severity by identifying cryptococcal nodules in the lungs and cryptococcal brain abscesses. For CM, lumbar punctures are commonly performed to test the patient CSF for the presence of *Cryptococcus* cells and to provide a measure of disease severity based on the opening pressure of the lumbar puncture, which is indicative of intracranial pressure. In addition, lumbar punctures are done to relieve high intracranial pressure by removing volumes of CSF. Increased intracranial pressure in CM has been hypothesized to be due to plugging of the arachnoid villi by clumping yeasts which disrupts normal CSF reabsorption and obstructs the outflow of CSF in the subarachnoid spaces resulting in increased intracranial pressure [93]. In humans, high fungal burden is commonly necessary to develop high lumbar puncture opening pressure, but is not the sole contributor [94]. In addition to fungal burden, the increase in intracranial pressure occurs in response to the immune system activation and cellular damage. Cellular damage causes an increase in reactive oxygen species while the release of matrix metalloproteinases, cytokines, and nitric oxide increase the permeability of the blood-brain barrier, alter cerebral blood flow, increase leukocyte receptors and binding. Together, this results in a change in the CSF composition and leads to the obstruction of CSF absorption and flow, neuronal damage, cerebral edema, and an increase of intracranial pressure. Aside from intracranial pressure, immune diagnostics such as CD4-T cell counts, white cell counts (lymphocytes and neutrophils), HIV-viral load, and more recently anti-GM-CSF antibodies are also common measures of clinical disease phenotypes that are associated with cryptococcosis [6,95]. In addition, many have reported the association between low CSF glucose concentration (<2.5 mmol/L) and low CSF/blood glucose ratio with poor clinical outcomes including death [96,97]. In the subsequent sections, we will discuss how cryptococcal genotypes and phenotypes affect the clinical presentation of disease.

## 4. Associations between Cryptococcal Genotypes and Clinical Presentation

As a preface to the following sections, it is important to clearly state that cryptococcal phenotypes vary by cryptococcal genotype. These differential phenotypic attributes can be observed in association with genotype from the broad genotypic relationship designations such as species, to the narrower designations such as molecular type and sequence type. In addition to phenotypic differences between strain genotypes, cryptococcal strain differences between the genetic sequences or gene expression might also contribute to pathogenicity through the adaptation of yeast cellular components, molecular function, and biological processes. Together, these attributes contribute to cryptococcal infection in specific populations, the clinical presentation of disease, and health outcomes (Table 2).

For example, broadly comparing the species complexes—*C. neoformans* primarily causes infections in immunocompromised individuals while *C. gattii* primarily causes infections in immunocompetent individuals [98,99]. However, under the umbrella of each species complex are individual species that have varying degrees of genetic diversity which endow them different phenotypic and physiological attributes that can result in deviation from the broader pathogenic trends. This can specifically be observed within the different lineages of the *C. gattii* species complex. As previously stated, *C. gattii* species complex is generally associated with causing infection in immunocompetent individuals, however in contrast to this generalization, VGIII and VGIV lineages primarily infect immunocompromised and HIV-infected individuals [98]. Furthermore, specific sequence types within the same lineage can display different phenotypes that equip one strain to cause infection in greater frequency or severity over another. These differences are clinically important. In this section, we will describe the phenotypic differences between each level of genotype designation from species complex to sequence type and detail how these cryptococcal attributes affect the various aspects of human disease (Table 2).

### 4.1. Mating Type, Species, and Serotype

Globally, the vast majority of invasive cryptococcal infections in humans are caused by strains within the *C. neoformans* species complex that are MATα, VNI, and serotype A [94,95]. *C. neoformans*, *C. deneoformans*, and *C. gattii* can all cause PC, CM, and skin lesions. *C. neoformans* more efficiently disseminates to the CNS and thereby more frequently causes CM. However, *C. gattii* primarily causes PC while *C. neoformans* and *C. deneoformans* primarily cause CM and *C. deneoformans* most often causes skin lesions [100]. Cryptococcal strains are generally either MATa or MATα. Strains of each mating type care capable of causing disease in humans and animals. The link between mating type and virulence in humans is convoluted due to extreme bias and overwhelming abundance of MATα clinical isolates. However, MATα strains are most prevalent among clinical isolates and, depending on fungal background, are often more virulent in animal models [29,101,102,103,104,105,106,107].

Interestingly, some interspecies hybrid strains can contain both mating type alleles. Unlike *C. neoformans* or *C. deneoformans* which are genetically haploid and display serotypes A or D respectively, the serotype AD hybrid can be genetically diploid and contain identical or different mating types. In mice, AD hybrid serotypes have attenuated virulence. Specifically, the genotype aADa was associated with decreased virulence compared to both αADα and aADα due to antagonistic epistatic interactions between the MATa alleles [108]. One human study reported that AD hybrid clinical isolates (67% αADa, 8% αADα, 16% aADα) have been reported to infect HIV-infected individuals and are associated with lower CrAg titers, reduced dissemination, less lung involvement, and improved CSF fungal clearance [109]. Another study found that AD hybrid serotypes are associated with significantly worse outcomes (death) compared to A and D serotypes which was thought to be due to the elevated melanin production and Th2 response [110]. In addition, AD hybrids have been found to have reduced capsule [110].

Considering serotypes A and D separately, serotype D may preferentially infect HIV-negative compared to AD hybrid strains. Among HIV-negative individuals, additional risk factors for serotype A infections include malignancy and other underlying disease while risk factors for serotype D infections include malignancy and no underlying disease [109]. Disease manifestation and clinical presentation can differ by serotype and results in different therapeutic approaches. For example, one study found that patients infected with serotype A and AD strains received induction combination therapy of amphotericin B and flucytosine more often than patients infected with serotype D [109]. Within the same study, a greater proportion of patients that were infected with serotype A strains exibited elevated intracranial pressure which indicates CNS involvment. In contrast, patients infected with serotype D presented with lower CrAg serum titers and less frequent abnormal brain imaging, suggesting that serotype D strains have reduced dissemination which results in less frequent CNS disease. Thus, patients that are infected with strains that cause CNS disease and symtoms are more likely to receive more aggressive antifungal therapy.

### 4.2. Molecular Type

Phylogenic studies using clinical isolates have found that the molecular types within the *C. neoformans* species complex, especially VNB, exhibit vast genetic diversity in comparison to *C. gattii* [22,111,112]. Now, with whole genome sequencing and GWAS, it is possible to utilize population and evolutionary genetics to dial down on the genetic changes in strains that are associated with disease compared to the genetic changes that have occurred only in response to exposure and survival within their environmental niche. For instance, a GWAS across cohorts of VNB isolates revealed gene sequence differences between clinical and environmental isolates among certain virulence factor and stress response genes [28]. Some of these genetic differences between molecular types may contribute to cryptococcal phenotypes and clinical presentation.

Among the *C. neoformans* species complex, strains within the VNI molecular type are phenotypically associated with Micro cells which can contribute to increased dissemination to the CNS. Supporting this notion, patients infected with VNI strains clinically present with vomiting and increased intracranial pressures [22].VNI and the VNB subtype, VNBI, are linked to increased capsule shedding which can affect the host immune response [22]. Interestingly, VNBI is more positively associated with fever compared to VNI. However, patients infected with VNBI strains are negatively associated with neck stiffness and diastolic blood pressures but are positively associated with lumbar puncture opening pressures (increased intracranial pressures). Like VNI, VNBI and VNBII are both also associated with lower CD4 counts which agrees with the general trend of *C. neoformans* causing infection in immunocompromised patients [22]. In addition to CM, patients with VNB infections also present with skin lesions and are associated with higher mortality [111]. VNII is associated with higher laccase production compared to both VNI and VNB clinical isolates as well as increased survival in ex-vivo CSF [111]. In addition, among a small cohort of 60 renal transplant recipients, nine patients infected with VNII strains had high survival rate compared to patients infected with strains of other molecular types [113]. This observation identifies a need for more robust studies to understand and characterize the relationships between cryptococcal molecular types and outcomes in transplant recipients.

Within the C. gattii species complex, both VGI and VGII disproportionately infect HIV-negative/immunocompetent individuals while VGIII and VGIV lineages primarily infect immunocompromised and HIV-infected individuals [98]. VGII is associated with high mortality among renal transplant patients in Brazil [113]. VGIV is positively associated with giant cells which are common in PC infections, higher CD4 counts and negatively associated with lumbar puncture opening pressures (e.g., decreased intracranial pressures), nausea, and vomiting [98].

### 4.3. Sequence Type

MLST analysis segregates cryptococcal isolates into numbered STs based on the genetic sequences of CAP59, GPD1, IGS1, LAC1, PLB1, SOD1, and URA5 [26]. Individual STs and groups of different STs have been associated with cryptococcal phenotypes, mortality, and other clinical parameters of disease. Initial evidence of a relationship between host mortality and groups of different STs was observed among cryptococcal isolates collected during the Cryptococcal Optimal ART Timing (COAT) trial. The COAT trial clinical isolates of different STs were categorized into three “virulence groups” based on survival time in humans and animals: (1) high virulence (ST93, ST40, ST31); (2) intermediate virulence (ST5, ST77, ST93); and (3) low virulence (ST5, ST40, ST31) [114]. These initial ST groupings were made using the phenotypic mortality data. Importantly, the inclusion of ST93 strains in both high virulence and intermediate virulence groups, as well as ST5 strains in both intermediate virulence and low virulence groups highlighted the complexity and convoluted relationship between the genetic sequences of the genes used for MLST, ST, and virulence in vivo and suggests there unknown contributors to virulence.

Further studies would show that differential clustering of STs by severity of virulence phenotype is linked to an evolutionary divergence between the genetic sequences of clinical isolates [110]. For instance, clustering genetically similar clinical isolates into nonredundant evolutionary “burst groups” have also identified an association between ST and mortality, as well as ST and cryptococcal virulence phenotypes. Among these genetically clustered burst groups of clinical isolates, Wiesner et.al. found that what they defined as Burst group 1 (ST1, ST36, ST 74, ST107, ST122) and Burst group 2 (ST11, ST29, ST40, ST45, ST76) were associated with high patient mortality while Burst group 3 (ST18, ST21, ST30, ST54) had greater patient survival [110]. In addition, they found that Burst group 1 was also associated with the cryptococcal virulence phenotype of increased capsule shedding [110]. Taken together, these findings demonstrate that there are associations between virulence (cryptococcal phenotype and host mortality) and groups of different STs that are genetically related. Individual STs have also been associated with mortality and clinical parameters of disease. Associations between patient outcomes and several STs have been reported in the literature, here we will discuss the associations found among two specific STs: ST5 and ST93.

*C. neoformans* VNI ST5 strains can cause infections in both HIV-infected and HIV-negative patients and are found in China, Vietnam, South America, Thailand, Korea, Laos, and USA [106,115,116,117,118,119,120,121,122,123,124,125,126,127,128,129,130,131,132,133,134]. In China and Vietnam, cryptococcosis is predominately caused by ST5 strains [116,119,121,123,124,133]. Among HIV-negative patients from China and Vietnam there is little diversity between the STs of clinical isolates and a predominance of ST5 cryptococcal infections [119]. Specifically, China has reported that a majority of their CM cases (most ST5) occur among individuals that are HIV-negative (71–96%) [121,123,124,133]. Among the HIV-negative cases 33–46% have no underlying diseases, 6–12% have hepatic diseases, 11–50% have autoimmune disorders or otherwise immunocompromised conditions, and 3–6% have chronic kidney disease [92,121,124]. In contrast, only 5–29% of CM cases occur in patients with HIV/AIDS [92,121,124]. HIV-infected patients from China and Vietnam often display greater cryptococcal ST diversity among their isolates but ST5 is still a highly prevalent ST [119].

Clinical presentation of cryptococcosis caused by ST5 strains differs by HIV status. Among HIV-negative individuals with no underlying disease, there were significantly more patients with cryptococcosis caused by ST5 that presented with stiff neck and vision disturbances compared to non-ST5 infections [124]. Furthermore, both HIV-infected and HIV-negative ST5 infected patients presented with CM (64% CM in HIV-infected, 61% in HIV-negative) [121]. However HIV-negative ST5 infected individuals also presented with PC (24%) and cryptococcosis at other infection sites (3%) [121]. Clinical presentations among HIV-infected individuals with ST5 genotype CM included increased lymphadenopathy, higher blood lymphocyte counts, higher CD4 counts, and lower initial CSF fungal burden [119].

Among patients with ST5 genotype cryptococcal infections, there have been mixed reports regarding associations with poor clinical outcomes such as death and disability which may be affected by HIV status, underlying condition, and even ethnicity. For example, one study from China found that mortality was significantly greater among HIV-infected (29%) to HIV-uninfected (14%) individuals with ST5 cryptococcosis [121]. Similarly, another study from China found that ST5 infections were associated with significantly greater mortality among patients with autoimmune disorders and, though not statistically significant due to small sample size, this study also had greater mortality among HIV-infected individuals [124]. Finally, a study from Vietnam showed that ST5 cryptococcal infections were associated with increased disability among CM survivors [119]. On the other hand, ST5 HIV-infected patients from the African COAT trial were associated with reduced mortality in both humans and mice [114]. In agreement, a study using Vietnam clinical isolates found that ST5 is not associated with greater mortality in a mouse inhalation model [131].

Though these clinical outcome ST5 data are limited and more information is required, one might hypothesize that individuals of Chinese or Vietnamese descent may be more susceptible to ST5 strains and may be more prone to poor clinical outcomes compared to individuals of African descent. In regards to ST specific cryptococcal phenotypes, ST5 cryptococcal cells have significantly increased variability in cell sizes and capsule sizes but no difference in urease activity, melanin formation, or population doubling time [22,127,131]. A GWAS comparing genetic sequences of ST5 to non-ST5 strains have identified genes that are associated with ST5 (more virulent phenotypes) and non-ST5 (less virulent phenotypes) [119].

ST93 is found in Africa, Brazil, Thailand, India, Colombia and is associated with high (early) mortality in humans and in mouse models [105,110,114,122,135,136]. Specific to patients infected with ST93 strains there is a positive association between mortality and CSF white cell count at diagnosis [114]. Additionally, HIV-infected individuals with CM that have deferred ART have higher CSF fungal burden [114]. Interestingly, mortality among ST93 infections is not associated with CSF fungal burden, but rather the interaction between host immune response and fungal virulence factors (capsule and melanin) [110]. As a result of a GWAS, ST93 specific genes were identified that were associated with virulence, human survival, antifungal susceptibility, and virulence in animals [137]. There are many more STs that are clinically relevant but ST5 and ST93 are representative of the role that ST has in the context of clinical disease presentation and mortality.

Taken together, it is clear that MLST analysis was an important contributor to our initial understanding of the relationship between genetic sequences, virulence phenotypes, and clinical disease. The inconsistencies between studies and contradicting findings indicate that there are more disease contributors aside from the differences in the genetic sequences of the genes that are used to classify ST. In this sense, ST represents the starting point in understanding the contribution of genetic sequences to pathogenicity and, in situations where whole genome sequencing is not feasible, MLST is still useful to identify associations. Going forward, whole genome sequencing and GWAS will clarify these convoluted relationships between *Cryptococcus* and humans in the context of disease. Furthermore, GWAS have only begun to shed light on the genes and genetic variations that contribute to cryptococcosis, but the present overlap among the identified genes with previous outcomes and from single gene mutants in animals suggests that we are on the right path to a better understanding of the host-yeast interactions at the human level.

## 5. Associations between Cryptococcal Phenotypes and Clinical Presentation

The classical cryptococcal virulence phenotypes have a demonstrated role in pathogenicity and mortality in animal models. However, their roles in the context of human infection are not fully understood. Clinical and environmental isolates are often labeled as either hypervirulent, intermediate, hypovirulent, or avirulent based on the quantity, combination, or degree of virulence that they display in vitro or in animal models. Virulence is measured in a spectrum that relates to how well the fungal virulence phenotypes contribute to yeast survival when exposed to stressful incubation conditions (drugs, high/low temperatures, pH, H_2_O_2_, limited nutrients, etc.) and how these factors contribute objectively to yeast proliferation, dissemination, and mortality in animals.

Going forward, it is important to note that cryptococcal expression of virulence phenotypes in vitro does not necessarily correlate with their expression in humans or in all animal models. Additionally, expression of virulence factors also varies by fungal background [138]. Clinical isolates that are clearly pathogenic in humans can be categorized as “avirulent” by the definitions of in vitro phenotyping due to the disconnect between in vitro conditions and the complex environment of the human host. Virulence phenotypes are dynamic and vary in response to the environment that the yeast inhabits [139]. Thus, virulence phenotype is site-specific which means virulence phenotypes among CSF isolates can differ from pulmonary isolates; both of which can differ from what is observed in vitro. Furthermore, in both human and animal studies there is evidence that mortality does not always correlate with the initial fungal burden/inoculation dose or other in vitro virulence phenotypes such as capsule size, phospholipase activity, melanin formation, proteinase activity, and antifungal susceptibility.

Overall, the inconsistencies between in vitro findings and in vivo animal models or patient outcomes suggests that there is more to cryptococcal virulence than just the known virulence factors and current in vitro conditions do not sufficiently replicate the host environment [42,111,114]. Although the precise phenotypic endpoint for judging virulence composites remains uncertain, it is encouraging that animals can replicate some of the disease production features observed in humans [42,114]. Our understanding of the role virulence phenotypes play in human infection might be improved by phenotyping cryptococcal strains directly from human or animal infection sites. For instance, the ability to perform RNAseq and create transcriptional profiles on yeast cells obtained directly from the human subarachnoid space has allowed identification of the genes and their networks important to yeasts under stress at the site of human infection [140]. Taken together, these data speak to the complexity of cryptococcal infection. Moreover, these data emphasize the importance of studying strain specific genotypes and phenotypes directly from humans to better understand the yeast virulence and fitness attributes required for pathogenicity in humans.

### Cryptococcal Virulence Phenotypes and Clinical Presentation

**Figure 3:**
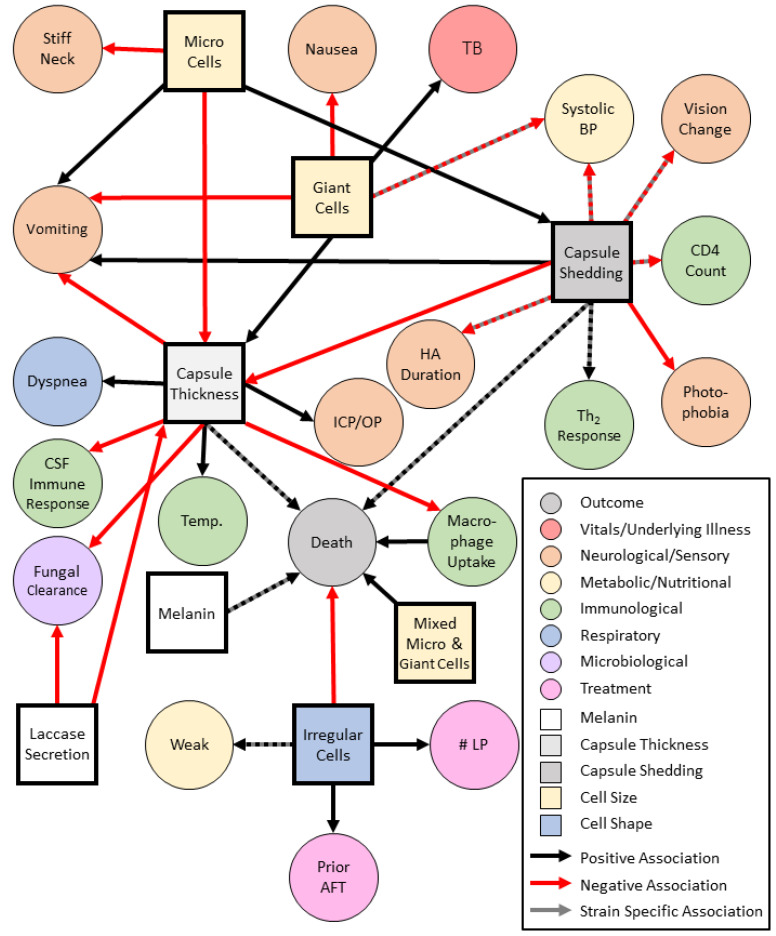
Cryptococcal Phenotype Associations with Human Clinical Presentation. Abbreviations: Tuberculosis (TB), Blood Pressure (BP), Headache (HA), Temperature (Temp.), Lumbar Puncture (LP), Cerebral Spinal Fluid (CSF), Intracranial Pressure/Opening Pressure (ICP/OP), Antifungal Therapy (AFT).

A prime example of the complex relationships of phenotype and disease manifestations is capsule and cell size. The cryptococcal polysaccharide capsule is essential for pathogenesis in humans. In mice, the size of the polysaccharide capsule is organ specific and is thickest in the lung over the brain, and is thinnest under in vitro conditions [139]. In humans, capsule thickness is positively associated with giant cells and negatively associated with Micro cells [22]. Cryptococcal cell size can vary by cryptococcal species and infection site. Giant cells have been shown to occur more frequently within the *C. gattii* species complex VGIV lineage (Table 2). Conversely, micro cells are not seen in VGIV strains. Lineages within the *C. neoformans* species complex are significantly associated with the Micro cell phenotype which is suggested to contribute to cryptococcal dissemination from the lungs to the CNS but are also capable of producing the giant cell phenotype. Micro cells are also associated with increased capsule shedding. *C. neoformans* isolates that produced Micro cells and Giant cells were positively associated with death (Figure 3) [22].

In the context of disease presentation, among the *C. neoformans* species complex, capsule thickness is positively associated with increased body temperature and shortness of breath and negatively associated with vomiting. In addition, shed capsule is negatively associated with systolic blood pressures, headache duration, photophobia, and visual changes which may be due to host immune responses and CNS disease [22]. Capsule shedding is not associated with increased intracranial pressure or lumbar puncture opening pressures but is positively associated with vomiting and mortality [22,44,110]. Although in vivo experiments have conflicting results, capsule size has been suggested to be associated with increased intracranial pressure, slow fungal clearance on treatment, and low CSF inflammation including low CSF white blood cell count, IL-4, IL-6, IL-7, IL-8, and interferon-gamma [44].

Irregular cell shapes are negatively associated with death and are thought to have reduced fitness [22]. Strains with greater laccase activity and high levels of fungal uptake by macrophages are associated with reduced fungal clearance during antifungal therapy with amphotericin B [45]. Specifically, hypocapsular cryptococcal strains are associated with greater laccase activity, high patient CSF fungal burden, increased uptake by macrophages in vitro, and reduced patient survival [45]. In agreement with clinical findings, *C. neoformans* has been associated with increased macrophage uptake but reduced intracellular proliferation compared to *C. gattii*, which was associated with high CSF fungal burden and reduced survival in animal models [141]. Furthermore, increased intracellular yeast replication in certain *C. gattii* strains is thought to be related to the upregulation of mitochondrial genes [142].

## 6. Associations between Human Attributes and Cryptococcosis

### 6.1. Associations between Human Genetics and Cryptococcosis

#### 6.1.1. Mannose-Binding Lectin

Mannose-binding lectin (MBL) is a pattern recognition protein that is a component of the innate immune system that binds microbial surface carbohydrates to assist in opsonophagocytosis and activation of the lectin complement pathway immune response. Previously, MBL has been associated with increased disease susceptibility to a variety of microorganisms, however its role in cryptococcosis remains unclear [143]. In China, a study that compared 42 HIV-negative patients with CM and 13 with PC found that patients with CM had higher CSF levels of complement C1q, factor B, MBL, sC5b-9, and factor H compared to PC controls. The same study showed a positive correlation between levels of total CSF protein and MBL, C1q, or factor B [144]. Furthermore, another study of HIV-negative CM patients of Chinese Han ethnicity, found an association between MBL deficiency and CM in which patients with the MBL O/O genotype were over four-times more likely to have CM [145]. In contrast, an Australian study analyzed MBL function, levels, and *MBL2* genotypes and determined for 36 HIV-negative patients with cryptococcosis had no association between MBL and cryptococcosis [146]. Congruently, a Thailand study of HIV/AIDS-infected individuals did not find an association between MBL levels or genotypes and cryptococcosis [147]. The differences in results between geographic areas may be due to the role of regional cryptococcal genotypes and phenotypes or these differences might simply be undetected due to low number of patients in these studies and lack of robust power to detect genetic associations.

#### 6.1.2. Immunoglobulins

Genetic differences among genes important for human immune response are another mechanism that may contribute to genetic predisposition to developing cryptococcal infections and poor clinical outcomes. The balance of immunoglobulins, IgM, IgG, and IgA are involved in cryptococcal immune response. X-linked hyper-IgM immunodeficiency syndrome is caused by mutations that occur in the CD40 ligand gene which results in decreased levels of IgG and IgA and elevated IgM has been shown to increase susceptibility to infections including invasive and cutaneous cryptococcosis particularly observed in pediatric male patients [148,149,150,151,152,153,154,155]. In addition, in vitro assays using *C. neoformans* found that IgM but not IgG inhibited Titan cell formation, reduced capsule thickness, and decreased the expression of chitin synthetase genes (*CHS1*, *CHS2*, *CHS8*, α-1–3-glucan synthetase (*AGS1*), and β-1,3-glucan synthetase (*FKS1*) [156]. Furthermore, IgM reduced the expression of the stress response pathways RIM101 and HOG1 [156]. There have been several studies suggesting a link with polymorphism in the Fc-gamma receptors of immunoglobulin genes and cryptococcosis [157,158,159].

#### 6.1.3. Dectin-2, Pentraxin-3, Interleukin

Dectin-2 is a dendritic cell-associated C-type lectin receptor involved in fungal pattern recognition during host immune response to pulmonary infection which is encoded by the gene CLEC6A on chromosome 12 of which there are three genotypes: CC, CT, and TT [160,161,162]. Interestingly, the genotype distribution is quite different between Europeans (40% CC, 60% CT, 0% TT), Africans (4% CC, 28% CT, 68%TT), and Asians (19–24% CC, 50–55% CT, 24–31% TT) [160]. A study among Chinese HIV-negative individuals with cryptococcosis found a significant difference between CT versus CC + TT groups indicating an association between Dectin-2 genotype and susceptibility to PC [160]. Pentraxin-3 (PTX-3) is another component of the human innate immunity response that is expressed by immune cells such as neutrophils, monocytes, dendritic cells, and endothelial cells [163]. Recently a study among Chinese HIV-negative individuals found that the PTX-3 plasma levels were significantly higher among individuals with the AA genotype compared to GA or GG genotypes [164]. In comparison to the GG genotype, AA was associated with increased risk (OR = 2.579; 95% CI = 1.202–5.535, *p* = 0.015,) of cryptococcosis in comparison to the GG genotype [164]. Furthermore, in the HIV-negative immunocompetent patients, the AA genotype had an even greater odds of developing cryptococcosis (OR = 4.399; 95% CI = 1.745–11.088, *p* = 0.002) [164]. In contrast, another study has found that the AA genotype was associated with a decreased risk of PC (OR = 0.37; 95% CI = 0.14–0.92; *p* = 0.037) [165]. More research is required to fully understand how genotype and polymorphisms within Dectin-3 and PTX-3 contribute to cryptococcosis. Finally, a single case of cryptococcosis in a child with a mutation in the IL-12RB1 gene demonstrates the importance of signaling pathways to clear intracellular pathogens [166].

#### 6.1.4. Anti-Granulocyte-Macrophage Colony-Stimulating Factor Autoantibodies

Part of host immune response, Granulocyte-Macrophage Colony-Stimulating Factor (GM-CSF) is a cytokine that stimulates peripheral blood mononuclear cells resulting in anti-cryptococcal activity [167,168,169]. Anti-Granulocyte-Macrophage Colony-Stimulating Factor (Anti-GM-CSF) is an anti-cytokine autoantibody that interferes with GM-CSF activity that has recently been implicated as a cryptococcosis risk factor among immunocompetent individuals and has been observed in both children and adults [95,170,171]. Anti-GM-CSF has specifically been associated with CM caused by members within the *C. gattii* species complex and not *C. neoformans* [6,172]. Detecting Anti-GM-CSF antibodies has the potential to be a novel means of identifying at-risk individuals to aid in therapeutic approach, healthcare planning and public health efforts [173,174].

#### 6.1.5. Biological Sex and Hormones

Cryptococcosis disproportionately occurs in males (61–80%) [96,124,175]. The role of host biological sex in cryptococcosis has historically been poorly understood, but recent studies are beginning to shed light on this topic and have been reviewed in detail [175]. There are sex-specific differences related to cryptococcal phenotype, growth rate, and host phagocytic activity [176]. Specifically, cryptococcal clinical isolates taken from females have slower growth rates and greater capsule shedding in the presence of testosterone but not 17-β estradiol [176]. From the host perspective, macrophages isolated from females are able to phagocytose yeast better than macrophages isolated from males resulting in lower fungal burden and increased fungal clearance [176]. In addition, compared to females, peripheral blood monocytes (PBMCs) isolated from males have greater proliferation of *C. neoformans* cells within them and during infection; male PBMCs had lower levels of CD3+, CD4+, and CD8+ T cells [177]. Furthermore, in the context of cryptococcal virulence factors, testosterone has been shown to increase the rate of melanin formation in *C. neoformans* cells in comparison to estradiol which may offer an added yeast benefit for pathogenesis [178].

### 6.2. Associations between Human Phenotypes and Cryptococcosis

In the previous sections, we have discussed the common underlying conditions that are associated with cryptococcosis in immunocompromised and immunocompetent populations. Furthermore, we have identified some of the potential human genetic factors that may predispose both HIV-infected and HIV-negative individuals to cryptococcal infections. Here we discuss clinical presentation of pediatric cryptococcosis and present illicit drug use as additional factors that may also influence development of disease.

#### 6.2.1. Pediatric Cryptococcosis

Cryptococcosis in the pediatric population is interestingly more uncommon than adults but, like adults, a majority of patients are male (55–70%) with CM caused by *C. neoformans* strains that are VNI/MATα [123,179,180,181]. Though some of these children may be HIV-infected (12–96%) or have other immunocompromising conditions (lupus 8%), certain geographic areas such as China (77–80%) or Colombia (46%) see cryptococcosis in children with no underlying illness [123,179,180,181]. Common symptoms are fever, cough, headache (significant association with CM), nausea and vomiting [181,182]. Poor clinical outcomes in children (i.e., disability and death) tend to be due to neurological symptoms or complications such as headache (significantly associated with CM), stiff neck, hydrocephalus, blindness, and seizures. [180,182]. One study from China found that among children with no underlying disease, 96% of them had PC but only 10% exhibited respiratory symptoms [182]. Other unique clinical parameters of pediatric cryptococcosis include elevated erythrocyte sedimentation rate, elevated CrAg titers, and elevated IgE and eosinophil levels which some have suggested may be related to *STAT3* Hyper IgE Syndrome [182,183,184,185,186]. Finally, the pediatric population in South Africa, boys were significantly more likely to be infected with ST8 (*C. neoformans*, VNI, MATα) compared to girls [179].

#### 6.2.2. Illicit Drug Use

Risky behaviors such as illicit drug use and unsafe sexual practices are associated with the acquisition of HIV which, in turn, is associated with developing cryptococcal infections. However, there is some evidence that illicit drug use alone or in conjunction with HIV, may also contribute to the development of cryptococcosis. Related to immunoglobulin defects, the use of methamphetamine (METH) has been shown to impair IgG-mediated phagocytosis and killing of cryptococcal cells in murine models while also facilitating intracellular replication [187,188]. In addition, murine models have also shown that METH enhances PC infection and dissemination to the CNS through alteration of cryptococcal virulence factors (e.g., capsule composition and capsule shedding) and by reducing the integrity of the host blood brain barrier [189,190]. Like METH, cocaine use has also been linked to alteration of the blood-brain barrier and might also be a risk factor for developing cryptococcal infections but further investigation is required to validate this hypothesis [191]. Furthermore, injection drug use in HIV-negative individuals has also been suggested to be a risk factor for cryptococcosis and poor clinical outcomes as a result of delayed diagnosis [192].

## 7. Conclusions

In this review, we attempted to frame the issues around the current understanding of the human fungal pathogen, *Cryptococcus*, in the context of human disease. Throughout this review, it is made evident that there are some conflicting results and associations between clinical outcomes and cryptococcal attributes. These inconclusive associations may both be correct, but for different reasons that are currently undiscovered. This is the challenge future research must aim to address and untangle. In the last two decades, there has been an expansion in the number of studies focused on cryptococcal molecular pathogenesis, providing a great platform to support our understanding of the genetic and phenotypic factors of this encapsulated yeast and how these factors relate to human disease. The power of genomic sequencing, phenotyping analysis, and well described clinical cohorts have truly started to expand our understanding of the mechanisms of cryptococcal disease. We now have the tools with which to make predictions from the genetic sequences of the fungus and host. As this review clearly shows, we are still in our infancy of a proper knowledge base, and our current understanding of the interplay between fungal-host phenotypes and genotypes is unclear. Thus, there is still more research that must be done and, fortunately, the trends and associations that have been identified so far are exciting to follow.

The ability of the yeast to produce disease is clearly complex and nuanced. *Cryptococcus* has a dynamic adaptability and plasticity to its genome which will provide challenges. Therefore, it will take careful studies strengthened by precise genomic data for large patient cohorts with linked clinical isolates and robust detail among clinical information to increase our understanding of the complex interaction between *Cryptococcus* and humans. In addition, it will take ingenuity to identify novel in vitro conditions that better replicate the human body so we can find reliable associations between virulence phenotypes or antifungal susceptibility with clinical outcomes. It will take great effort and scientific collaboration but, it is realistic to predict that in the future, we may be able to breakdown the genetics of the fungus and the host to make predictions for disease outcomes. It is also realistic to expect that from a certain genotype or phenotype of the isolate, we may be able to predict outcome in the host, or at least tailor a management regimen after identifying the fungal pedigree. Moreover, with greater amounts of detailed clinical information, including clinical tests outside of cryptococcal and HIV diagnostics, we might identify additional risk factors, signs, and symptoms that will allow clinicians to diagnose cryptococcal infections earlier, especially in immunocompetent individuals and those with pulmonary cryptococcosis. All C. neoformans and C. gattii strains are not created equal. We need to identify the attributes that distinguish the “bad-actor” from the “worse-actor” strains and, on the way to discovery, we will learn a lot about cryptococcosis and hopefully identify ways to improve clinical outcomes.

## Figures and Tables

**Table 1:** Genome Diversity in *Cryptococcus neoformans* and *Cryptococcus gattii* strains.

Strain	MAT	Species	Lineage	Serotype	Genome Size (Mb)	Source	Origin	Ref.
H99	α	*C. neoformans*	VNI	A	18.9	Clinical	USA	[27]
Bt63	a	*C. neoformans*	VNBI	A	18.4	Clinical	Africa	[28]
Bt1	α	*C. neoformans*	VNBII	A	18.3	Clinical	Africa	[28]
JEC21	α	*C. deneoformans*	VNIV	D	19.1	Lab Derived	Multiple ^1^	[29]
B-3501A	α	*C. deneoformans*	VNIV	D	18.5	Lab Derived	Multiple ^1^	[29]
WM276	α	*C. gattii*	VGI	B	15.8	Environment	Australia	[30]
E566	a	*C. gattii*	VGI	B	17.7	Environment	Australia	[30]
RU294	α	*C. gattii*	VGI	B	17.7	Environment	Africa	[30]
CBS10089	α	*C. deuterogattii*	VGII	B	17.0	Clinical	Greece	[31]
CBS1930	a	*C. deuterogattii*	VGII	B	17.4	Clinical	Greece	[30,32]
R265	α	*C. deuterogattii*	VGIIa	B	17.2	Clinical	Canada	[33,34]
RAM5	α	*C. deuterogattii*	VGIIb	B	17.3	Clinical	Australia	[30]
B8571	α	*C. deuterogattii*	VGIIc	B	17.1	Clinical	USA	[30]
NIH312	α	*C. bacillisporus*	VGIII	C	15.9	Clinical	USA	[35]
CA1873	a	*C. bacillisporus*	VGIII	B	17.4	Clinical	USA	[30,36]
CBS10101	α	*C. tetragattii*	VGIV	C	15.8	Veterinary	Africa	[30,31,37]
MF34	α	Unnamed	VGV	B	17.9	Environment	Africa	[25]

Abbreviations: Mating type (MAT), Megabases (Mb), Reference (Ref.) ^1^ Lab derived strains were made at University of California San Francisco (USA) from the ancestor strains NIH433 (Denmark, Environmental) & NIH12 (USA, Clinical).

**Table 2:** Cryptococcal Genotype Associations with Cryptococcal Phenotypes and Human Clinical Presentation.

Crypto. Genotype	Associations with Human Clinical Presentation	Assoc. with Crypto. Phenotype
Mating Type	Clinical Isolate Details: MATα > MATa	
Species Complex *C. neoformans* (Serotype A, D, AD)	Population: Immunocompromised> Immunocompetent, HIV-infected > HIV-uninfected PC Symptoms: Cough, Dyspnea, ↑Respiration Rate, Chest Pain, Fever CM Symptoms: Headache, Fever, Neck Pain/Stiffness, Nausea/Vomiting, AMS, Confusion, BC, Photophobia, VC, Seizure, Night Sweats, Limb Weakness,	
*C. neoformans*	Population: Immunocompromised Disease: CM > PC, Skin Lesions < (versus *C. deneoformans*) and > (versus *C. gattii*) Yeast Localization: CNS > Blood, Lungs, Skin, Other organs	
Serotype A	Population: Immunocompromised > Immunocompetent Risk Factors for HIV-negative Individuals: Malignancy, Other Immunocompromising Conditions Diagnostic Tests: ↑ICP/OP Antifungal Therapy: AmB + 5FC Induction therapy > (versus Serotype D)	
VNI (Serotype A)	Symptoms: ↑Vomiting, ↑ICP/OP Clinical Tests: ↓CD4 Count	Cell Size: Micro Cells, Normal Capsule: ↑Capsule Shedding
VNII (Serotype A)		Melanin: ↑ Laccase Production (vs. VNI or VNB), ↑Survival in ex-vivo CSF
VNB (Serotype A)	Disease: CM Symptoms: Skin Lesions Patient Outcome: ↑Death	
VNBI (Serotype A)	Symptoms: ↑Fever (versus VNI), ↑ICP/OP, ↓Neck Stiffness, ↓Diastolic BP, Clinical Tests: ↓CD4 Count	Capsule: ↑Capsule Shedding
VNBII (Serotype A)	Clinical Tests: ↓CD4 Count	
**Crypto. Genotype**	**Associations with Human Clinical Presentation**	**Assoc. Crypto. Phenotype**
VNIII (Serotype AD)	Population: Immunocompromised, HIV-Infected > HIV-Uninfected Clinical Isolate Details: αADa > aADα > αADα Yeast Localization: ↓Dissemination to CNS Symptoms: ↓Lung involvement, Diagnostic Tests: ↓CrAg Titers, ↑CSF Fungal Clearance, ↑Th_2_ Cytokine Response Antifungal Therapy: AmB + 5FC Induction therapy > (versus Serotype D) Patient Outcome: ↑Death	↑Melanin Production
*C. deneoformans* VNIV (Serotype D)	Population: Immunocompromised, HIV-Infected > HIV-Uninfected (vs. AD hybrid) Risk factors for HIV-Uninfected Individuals: Malignancy, No Underlying Conditions Disease: CM > PC, Skin Lesions > (versus *C. neoformans* or *C. gattii*) Diagnostic Tests: ↓Blood CrAg Titers, ↓Brain Abnormality (Radiology) Antifungal Therapy: AmB + 5FC Induction therapy < (versus Serotype A or AD)	
Species Complex *C. gattii* (Serotype B, C)	Population: Immunocompetent > Immunocompromised Disease: PC > CM, Skin Lesions < (versus *C. deneoformans* and *C. gattii*) PC Symptoms: Cough (Productive or Unproductive), Dyspnea, Chest Pain, Fever, Hemoptysis, Pulmonary Nodules/Mass Lesions (Patient can be asymptomatic) CM Symptoms: Headache, Fever, AMS, Confusion, BC, Neck Pain/Stiffness, Nausea/Vomiting, Photophobia, VC, ONS, Hearing Loss, Cerebellar Abnormalities, Limb Weakness, Seizure, Cranial Nerve Abnormalities, Papilledema	
*C. gattii–*VGI	Population: Immunocompetent, HIV-Uninfected	
*C. deuterogattii–*VGII	Population: Immunocompetent, HIV-Uninfected Patient Outcome: ↑Death (Renal Transplant Patients)	
*C. bacillisporus–*VGIII	Population: Immunocompromised, HIV-Infected	
*C. tetragattii–*VGIV	Population: Immunocompromised, HIV-Infected Disease: CM > PC Diagnostic Tests: ↑CD4 Count, ↓ICP/OP Symptoms: ↓Nausea, ↓Vomiting, ↑BP, Night Sweats, VC, Headache, AMS, Confusion, BC, Photophobia, Fever	Cell Size: Giant/Titan cells, Normal Size, No Micro Cells

Abbreviations: Associations (Assoc.), Pulmonary Cryptococcosis (PC), Cryptococcal Meningitis (CM), Central Nervous System (CNS), Intracranial Pressure (ICP), Opening Pressure(OP), Amphotericin B (AmB), Flucytosine (5FC), Altered Mental Status (AMS), Behavioral Changes, Vision Changes (VC), Optic Nerve Swelling (ONS).
